# Antegrade pancreatoscopy-guided laser recanalization of a completely occluded pancreaticojejunostomy anastomosis

**DOI:** 10.1055/a-2965-6168

**Published:** 2026-09-30

**Authors:** Masahiro Yamamura, Takeshi Ogura, Nobuhiro Hattori, Kimi Bessho, Junichi Nakamura, Hiroki Nishikawa

**Affiliations:** 1 Pancreatobiliary Advanced Medical Center 38588 Osaka Medical and Pharmaceutical University HospitalTakatsukiOsaka PrefectureJapan; 2 2nd Department of Internal Medicine 13010 Osaka Medical and Pharmaceutical UniversityTakatsukiJapan; 3 Endoscopy Center 38588 Osaka Medical and Pharmaceutical University HospitalTakatsukiJapan

Pancreaticojejunostomy anastomotic stricture (PJS) is a known adverse event after pancreaticoduodenectomy. Although balloon dilation and fully covered self-expandable metal stent placement have been reported, PJS may recur because the scar tissue itself remains. 1​ 2​ ​3​ Antegrade pancreatoscopy–guided laser dissection or ablation through an endoscopic ultrasound–guided pancreatic duct drainage (EUS-PD) route has recently been developed to remove scar tissue. 4​ ​5​ However, recanalization of a completely occluded anastomosis remains challenging. We present a case of successful antegrade recanalization using pancreatoscopy-guided laser ablation ( Video 1 ).

**Video 1** Antegrade pancreatoscopy–guided laser ablation through an endoscopic ultrasound–guided pancreatic duct drainage route enabled the successful recanalization of a completely occluded pancreaticojejunostomy anastomosis and placement of two plastic stents.


A 70-year-old man was admitted to our hospital for the treatment of PJS. He had undergone pancreaticoduodenectomy for ampullary carcinoma 2 years earlier and subsequently underwent EUS-PD for the PJS. During stent exchange, pancreatography revealed no contrast medium flow through the anastomosis into the jejunum, and a guidewire could not be advanced across it, suggesting complete occlusion ( Fig. 1 ). The plastic stent was exchanged for a metal stent. Two days later, antegrade treatment was attempted. A pancreatoscope was inserted through the metal stent, confirming complete anastomotic occlusion ( Fig. 2 ). Because a guidewire could not be advanced across the anastomosis, laser ablation was performed stepwise, beginning at the surface of the scar tissue and progressing gradually deeper ( Fig. 3 ). Compared with an approach from the small-bowel side, the antegrade approach allowed fluoroscopically visible gas in the jejunal lumen to serve as a landmark, facilitating ablation in the appropriate direction. During continued ablation, the metal stent was removed and the pancreatoscope was reinserted to allow further treatment. Contrast medium subsequently flowed into the jejunum ( Fig. 4 ), and the pancreatoscope could be advanced across the anastomosis, confirming successful recanalization. Finally, two plastic stents were placed ( Fig. 5 ).

**Fig. 1:**
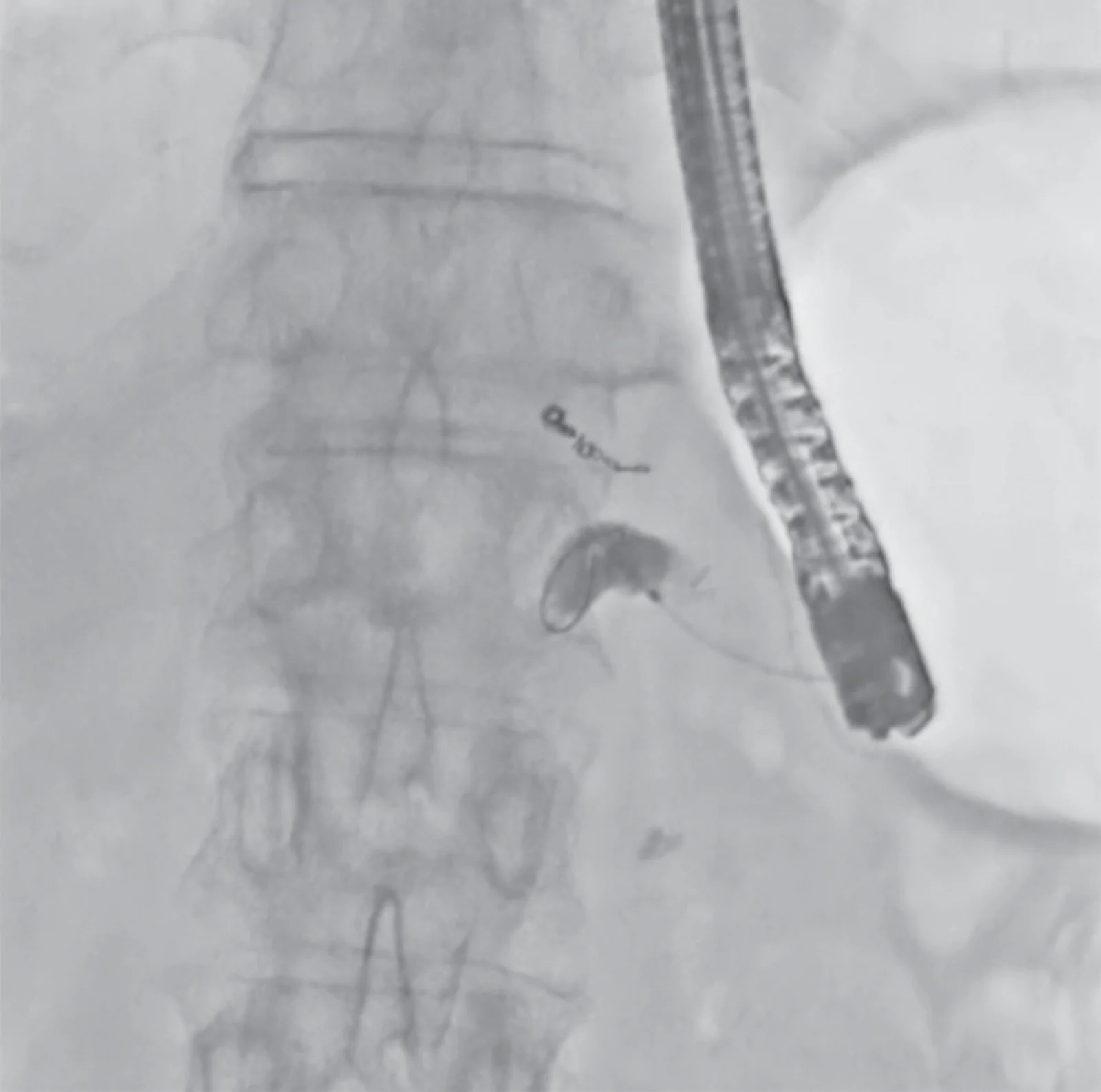
Pancreatography revealed no contrast flow through the pancreaticojejunostomy anastomosis into the jejunum.

**Fig. 2:**
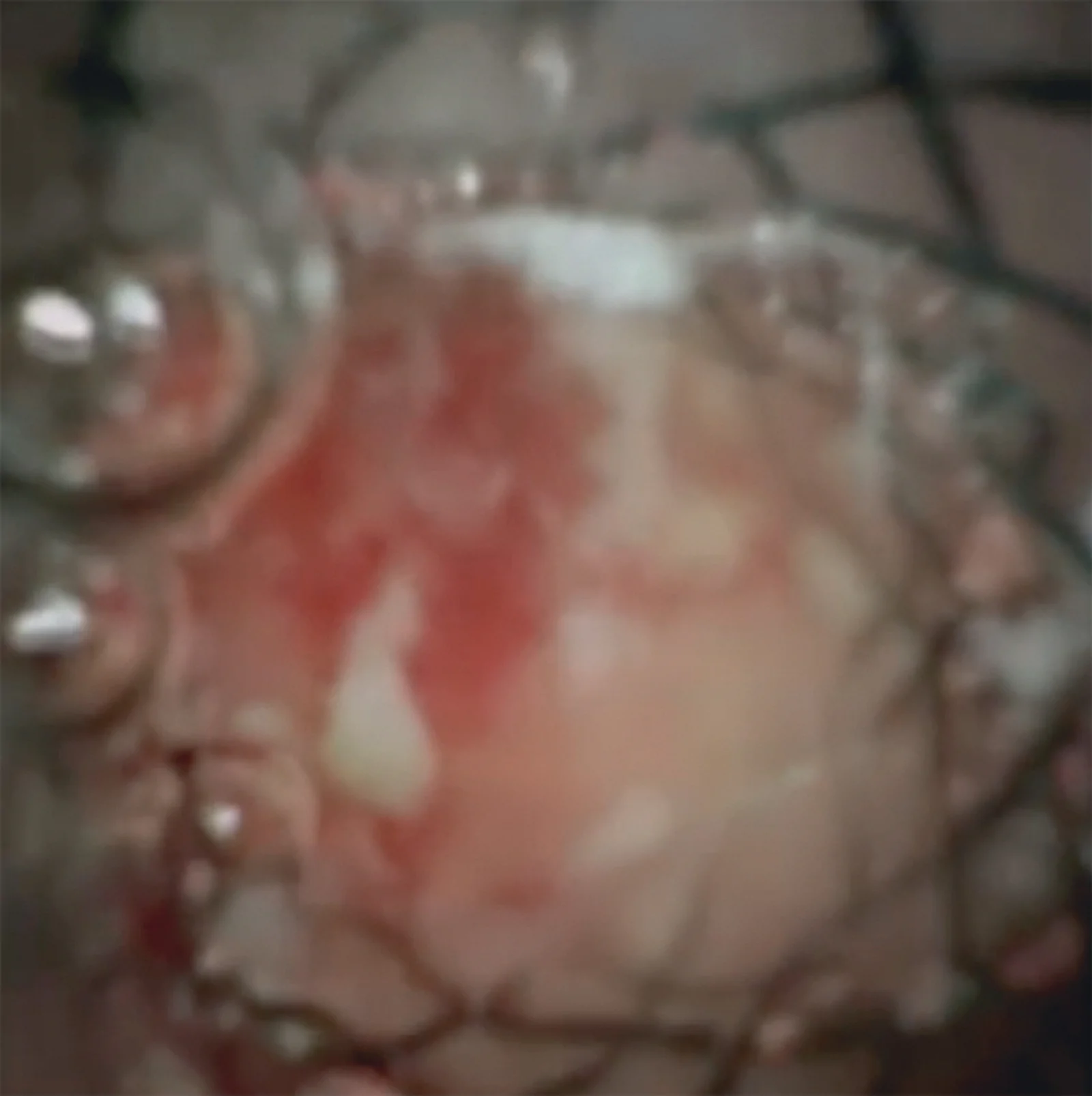
A pancreatoscopic image confirming the complete occlusion of the pancreaticojejunostomy anastomosis.

**Fig. 3:**
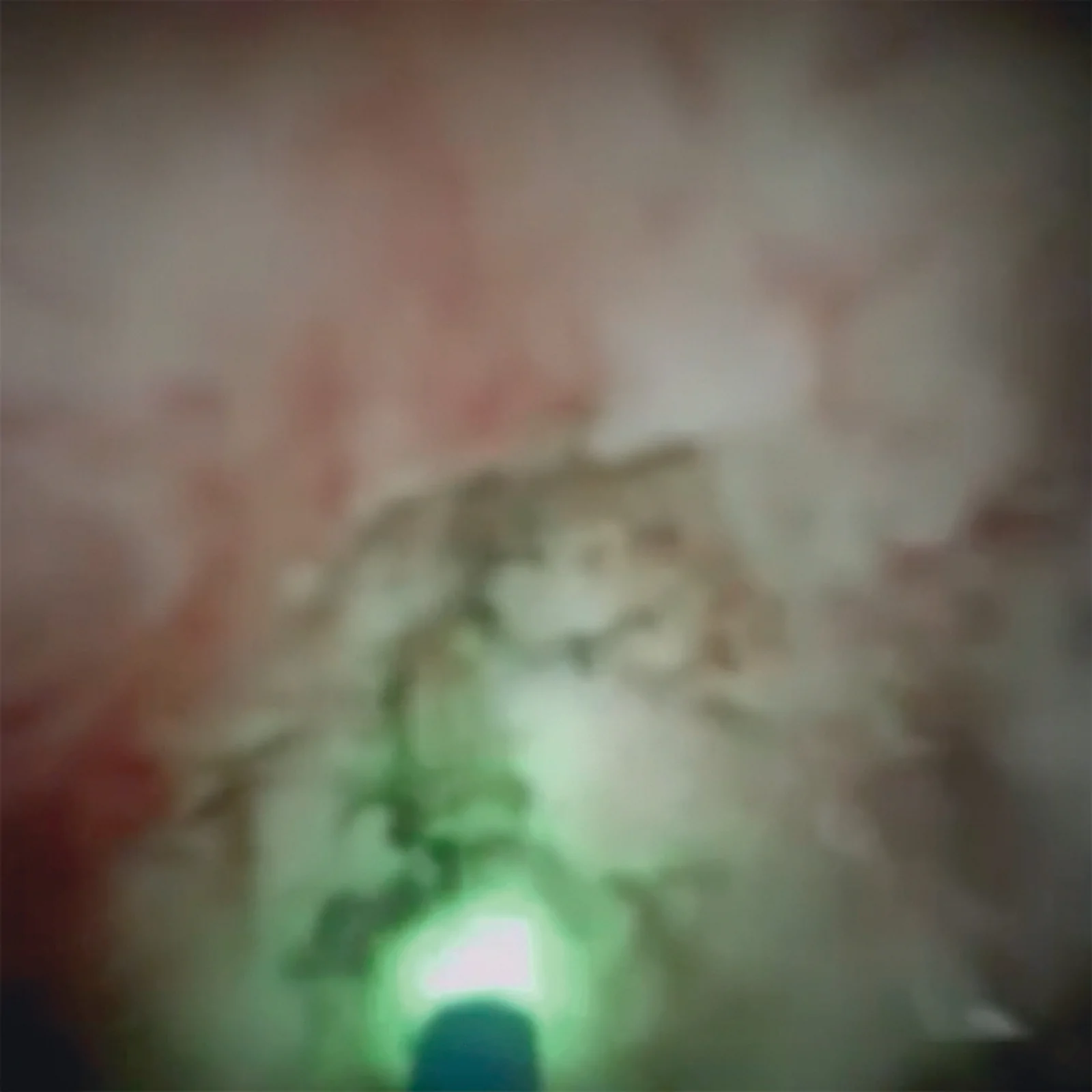
A pancreatoscopic image showing laser ablation of the scar tissue.

**Fig. 4:**
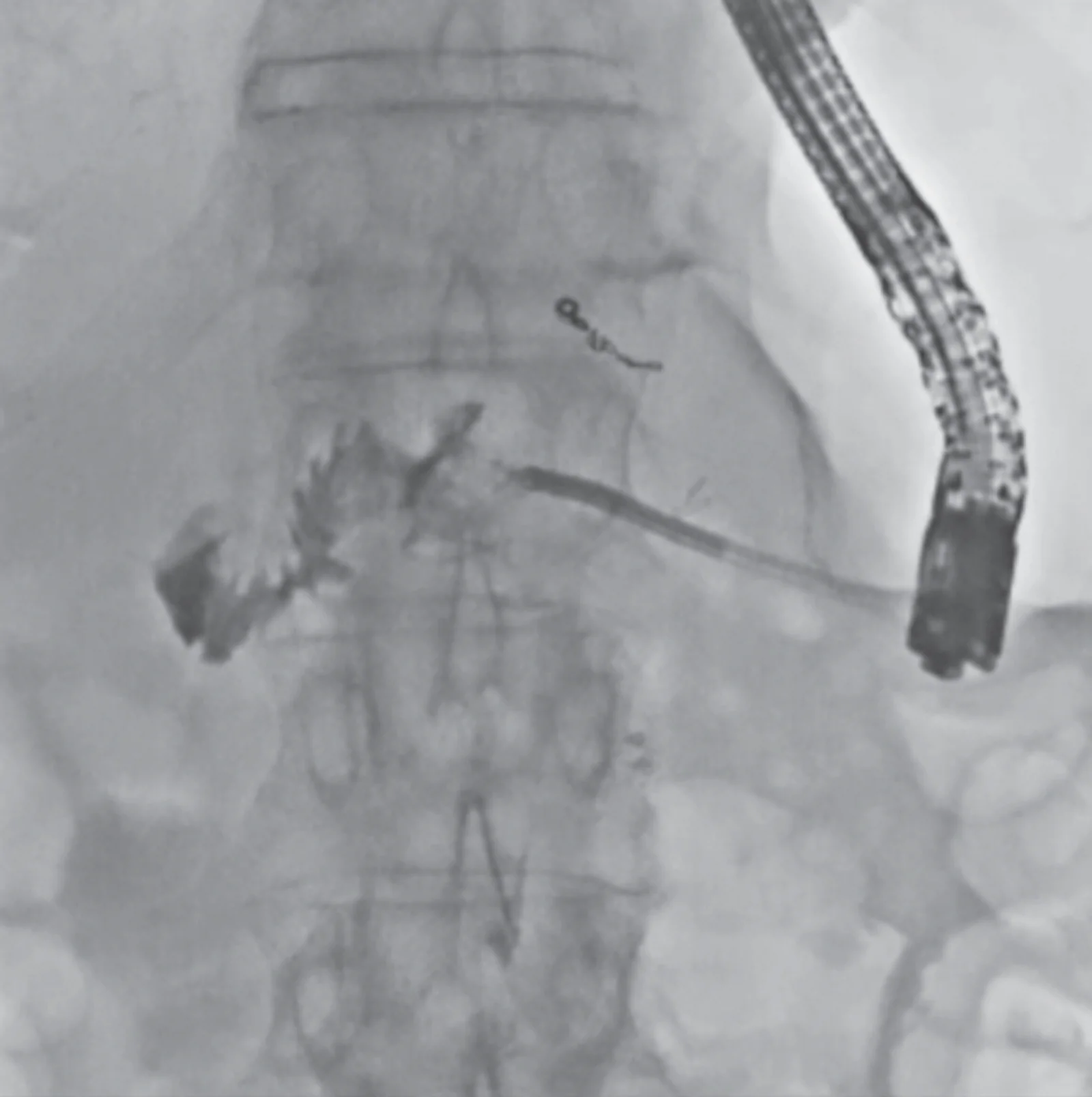
Pancreatography revealed contrast flow through the recanalized anastomosis into the jejunum.

**Fig. 5:**
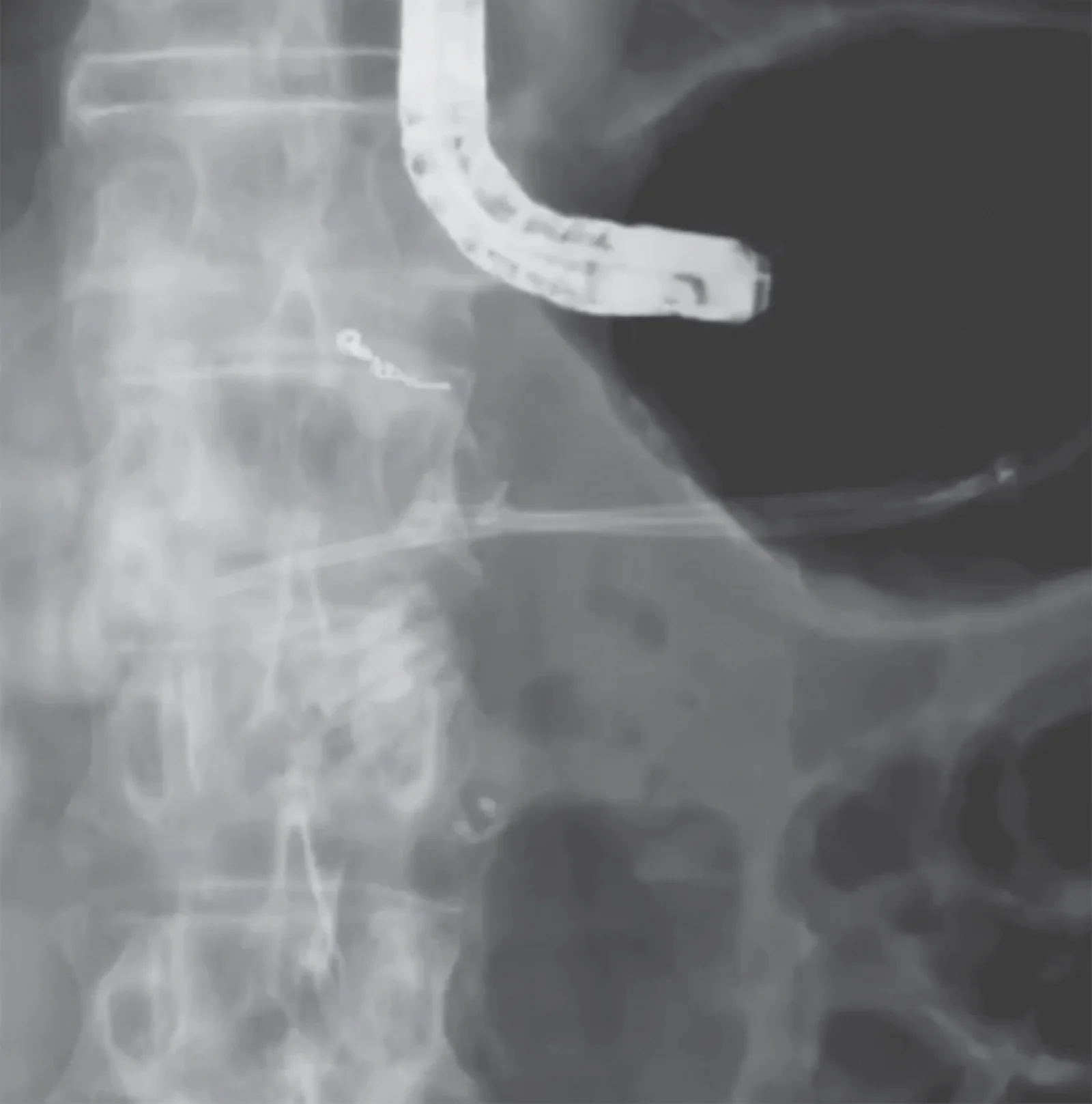
Two plastic stents were placed across the recanalized anastomosis.

Antegrade pancreatoscopy–guided laser ablation through an EUS-PD route may be a safe and effective option for complete pancreaticojejunostomy anastomotic occlusion, although further evaluation is needed.

Endoscopy_UCTN_Code_TTT_1AR_2AD

## Declaration of GenAI Use

During the preparation of this manuscript, the authors used ChatGPT (OpenAI) for English translation and language editing. The authors reviewed and edited the output and take full responsibility for the content of the manuscript.
